# Foraging Behavior of Two Pollen Wasp Species of the Genus *Celonites* Latreille, 1802 (Hymenoptera: Vespidae: Masarinae), from the Altai Mountains

**DOI:** 10.3390/insects14050408

**Published:** 2023-04-24

**Authors:** Alexander V. Fateryga, Volker Mauss, Maxim Yu. Proshchalykin

**Affiliations:** 1 T.I. Vyazemsky Karadag Scientific Station—Nature Reserve of RAS—Branch of A.O. Kovalevsky Institute of Biology of the Southern Seas of RAS, Nauki Str. 24, Kurortnoye, 298188 Feodosiya, Russia; 2 Entomology Department, Stuttgart State Museum of Natural History, Rosenstein 1, D-70191 Stuttgart, Germany; 3 Federal Scientific Center of the East Asia Terrestrial Biodiversity, Far Eastern Branch of the Russian Academy of Sciences, 100-Let Vladivostoku Ave. 159, 690022 Vladivostok, Russia

**Keywords:** flower-visiting behavior, morphology, palaearctic region, taxonomy, trophic relationships, vespid wasps

## Abstract

**Simple Summary:**

The pollen wasps are a fascinating group of insects that live similarly to solitary bees: females provision their brood cells with a mixture of pollen and nectar instead of insect prey. The relationships of these wasps with flowering plants are of special interest, since most species of the masarine wasps are specialized for particular groups of plants and/or flower types. Within the genus Celonites, trophic preferences are better known for the Afrotropical and the Mediterranean species, while they are nearly unknown for representatives from Central Asia. We studied two closely related species from the Altai Mountains: Celonites kozlovi and C. sibiricus. The first species is a generalist, which is not typical of the genus. Due to behavioral plasticity, it is able to use flowers of various plant species from at least five families (Asteraceae and Lamiaceae predominate) as pollen sources. The second species has specialized pollen-collecting structures that allow females to use flowers of Lamiaceae efficiently, while other plants are only occasionally visited. These adaptations are similar to those of some Mediterranean species of Celonites, but they evolved independently. The existence of two different foraging strategies by the closely related C. kozlovi and C. sibiricus leads to niche segregation and allows them to coexist in the same extreme habitats at the northernmost border of the range of the pollen wasps.

**Abstract:**

*Celonites kozlovi* Kostylev, 1935, and *C. sibiricus* Gusenleitner, 2007, coexist in semi-deserts of the Altai Mountains. The trophic relationships of these pollen wasp species to flowers are largely unknown. We observed the flower visits and behaviors of wasps on flowers; pollen-collecting structures of females were studied using SEM; the taxonomic position of these two species was ascertained with the barcoding sequence of the mitochondrial COI-5P gene. *Celonites kozlovi* and *C. sibiricus* form a clade together with *C. hellenicus* Gusenleitner, 1997, and *C. iranus* Gusenleitner, 2018, within the subgenus *Eucelonites* Richards, 1962. *Celonites kozlovi* is polylectic in the narrow sense, collecting pollen from flowers of plants belonging to five families (with the predomination of Asteraceae and Lamiaceae) using diverse methods for both pollen and nectar uptake. In addition, this species is a secondary nectar robber, which has not been observed in pollen wasps before. The generalistic foraging strategy of *C. kozlovi* is correlated with an unspecialized pollen-collecting apparatus on the fore-tarsi. In contrast, *C. sibiricus* is broadly oligolectic, predominantly collecting pollen from flowers of Lamiaceae. Its specialized foraging strategy is associated with apomorphic behavioral and morphological traits, particularly specialized pollen-collecting setae on the frons, which enable indirect pollen uptake using nototribic anthers. These adaptations in *C. sibiricus* evolved independently of similar specializations in the *Celonites abbreviatus*-complex. *Celonites kozlovi* is re-described, and males are described for the first time.

## 1. Introduction

The pollen wasps (subfamily Masarinae s. l., including Gayellini and Masarini) are one of the most fascinating groups of insects. They are not very species-rich, with only 374 species having been described so far [1]. Many of them are rare and poorly known insects, which are not frequently collected. Masarine wasps are distributed mainly throughout Mediterranean and temperate to hot semi-arid to arid regions outside the tropics, but not those further north than about 50° N or further south than 50° S [2,3]. They are particularly diverse in the Mediterranean area, Western and Central Asia, the south-west of North America, the south-west of Africa, and Australia. A few Neotropical genera occur in humid environments [4]. All species of pollen wasps studied so far live solitarily. After mating, each female builds a nest and provisions its brood cells with a mixture of pollen and nectar as a protein source; pollen and nectar are transported internally in the crop [2,3,5,6]. The pollen wasps are one of just two groups of the aculeate Hymenoptera, which have made a radical switch from insect prey to pollen and nectar as their main brood food; the second group is bees. A bee-like life form probably evolved within the stem lineage of Masarinae s. l. [7,8,9], although a molecular phylogenetic analysis indicates that it might have evolved independently within Gayellini and Masarini [10], and in the latter case, these tribes should be treated as separate subfamilies. The relationships of masarine wasps with angiosperm plants are of special interest [2,5,11,12,13], since most species of these insects are specialized for particular plant taxa and/or types of flowers, and some plants are even adapted to pollination by pollen wasps [3].

The genus *Celonites* Latreille, 1802, is distributed in the Palaearctic and the Afrotropical regions [3,5,14]. Currently, it contains 62 recognized species [15], making it the second-largest genus of the Masarinae after *Quartinia* André, 1884. Palaearctic species of *Celonites* are known to collect pollen from several genera of plants belonging to the families Lamiaceae and Boraginaceae, while the Afrotropical representatives are usually specialized in Scrophulariaceae and/or Campanulaceae and less often use Asteraceae, Boraginaceae, and Aizoaceae as either primary or secondary pollen sources [2,3,5,11,12,13,16,17,18,19]. In general, trophic preferences are better known for Afrotropical and the Mediterranean species of *Celonites*, while flower-visiting records for the Central Asian representatives are scarce.

The south-east of the Altai Mountains in Russia and the neighboring territories of Mongolia and Kazakhstan represent the northernmost border of the range of pollen wasps in Central Asia. Two species are known to occur in this area, which are *Celonites kozlovi* Kostylev, 1935, and *C. sibiricus* Gusenleitner, 2007 [20,21,22,23]. A preliminary study of their flower-visiting behavior has been conducted previously [23], and both species were found to be narrowly oligolectic, sensu Müller and Kuhlmann [24], collecting pollen exclusively from flowers of the genus *Dracocephalum* L. (Lamiaceae). However, the existence of an oligolectic association with Lamiaceae in *C. kozlovi* was recently questioned [18] because it does not correlate with any specialized pollen-collecting structures present in other species of the genus, including *C. sibiricus,* which are adapted to pollen uptake from nototribic flowers. Nototribic flowers are zygomorphic flowers with stamens and styles placed in such a manner that they come into contact with the dorsal surface of the pollinator’s body [25,26].

The purpose of the present contribution is to study the trophic relationships of *C. kozlovi* and *C. sibiricus* with their forage plants in detail, to describe their pollen-collecting structures, as well as to ascertain the taxonomic position of these two species. A brief re-description of the poorly known species, *C. kozlovi*, including a description of the hitherto unknown males, is also provided in the Appendix A and Appendix B.

## 2. Materials and Methods

### 2.1. Field Investigations

**Figure 1:**
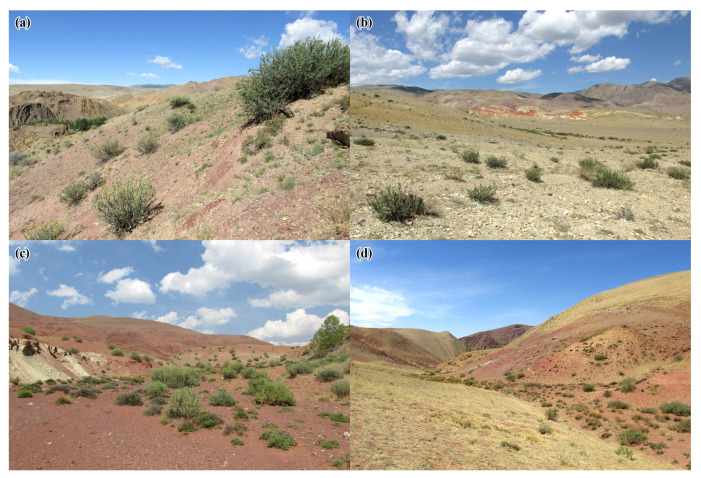
Localities in the Altai Mountains where *Celonites kozlovi* Kostylev, 1935, and *C. sibiricus* Gusenleitner, 2007, coexist: (**a**) Tydtuyaryk River valley; (**b**) Mars Natural Landmark; (**c**) 3 km to the north-east of Kokorya; (**d**) 5 km to the north-east of Kokorya.

The research was carried out in the Kosh-Agach District of the Altai Republic (Russia) in June 2022. Data were collected at four localities where *Celonites kozlovi* and *C. sibiricus* co-occur (Table 1). At all localities, the habitat consisted of a mountain semi-desert with sparse herbaceous and shrub vegetation (Figure 1). Observations took place in the morning (9:00–11:00, solar time in which 12:00 o’clock corresponds to the highest position of the sun) and during sunny weather. Wasp activity was observed visually and documented using a Canon EOS RP digital camera (Canon Inc., Ota, Tokyo, Japan) with a Sigma AF 105 mm f/2.8 macro lens (scale up to 1:1). Flower preferences of the wasps were studied by counting the number of sightings (=first observations) of flower-visiting individuals while walking randomly across the study stites. The total investigation time was 13 h (Table 1). Wasp specimens were selectively collected and deposited in the research collection of A.V.F.

All flowering plant species were photographed and voucher specimens were collected and preserved dried, regardless whether they were visited by the wasps or not. Plant specimens were deposited in the herbaria of the T.I. Vyazemsky Karadag Scientific Station—Nature Reserve of RAS—Branch of A.O. Kovalevsky Institute of Biology of the Southern Seas of RAS, Feodosiya, Russia (herbarium PHEO), and the M.V. Lomonosov Moscow State University, Moscow, Russia (herbarium MW) [27]. Plant species were identified with the help of the *Key to Plants of the Altai Republic* [28] and the Plantarium website [29]; photographs were also uploaded to the same website. We followed POWO [30] for the taxonomic treatment of the identified plants.

### 2.2. Morphological Investigations

Photographs of the collected specimens were taken with a Canon EOS 550D digital camera (Canon Inc., Ota, Tokyo, Japan) and a Yongnuo YN-14EX macro flash attached to an Olympus SZ60 stereomicroscope (Olympus Corporation, Shinjuku, Tokyo, Japan). Multifocus images were created from stacks of photographs using CombineZP 7.0 software (Alan Hadley). The final illustrations were post-processed for sharpness, contrast, and brightness using Adobe Photoshop CS2 9.0 software (Adobe Inc., San Jose, CA, USA). Male genitalia were extracted after re-softening the specimens, and were then boiled in 10% NaOH for 5 min. After that, they were rinsed in 80% ethanol, and only then, they were stored and studied in glycerin. The genitalia of two males of each species were studied. SEM micrographs of the wasp structures were taken using a Hitachi SE3500 Scanning Electron Microscope (Hitachi Ltd., Chiyoda, Tokyo, Japan). Three females of each species were studied. The wasp fragments were simply air-dried, mounted on stubs and coated with gold and palladium.

### 2.3. DNA Barcoding

DNA barcoding was accomplished by AIM Advanced Identification Methods GmbH Leipzig following standard methods of DNA extraction from a single leg of dry specimens or specimens collected and stored in 96% pure ethanol. The barcoding fragment of the gene Cytochrome Oxidase subunit 1 (COI-5P) was amplified via PCR cycle sequencing with forward and reverse strand and sequence editing. Nucleotide sequences of 32 individuals from 14 Palaearctic species of *Celonites* and 2 individuals of *Quartinia major* Kohl, 1898, were obtained. All nucleotide sequences were uploaded and stored in the BOLD database (www.boldsystems.org, accessed on 14 February 2023). In addition, COI-5P sequences of two individuals of *Celonites yemenensis* Giordani Soika, 1957, were provided by Christian Schmid-Egger and Stefan Schmidt of the GBACU-project. Another three public COI-5P sequences of *Ceramius micheneri* Gess, 1968, *Ceramius socius* Turner, 1935, and *Jugurtia braunsiella* (von Schulthess, 1930) provided by the BOLD database were added as additional outgroups to the data set (for individual BOLD process IDs and sequence lengths, see Figure 7; for further details, see Table S1 in the Supplementary Materials). A phenetic (similarity) method based on genetic distances was used for a crude assessment of the phylogenetic position of the studied species. A neighbor-joining BOLD TaxonID Tree was computed, including 39 barcoding fragments of COI-5P sequences of variable length, using the following parameters: distance model Kimura 2 Parameter; pairwise deletion of positions containing gaps and missing data; minimum complete overlap 0 bp; alignment with BOLD Aligner (Amino Acid based HMM); individual nucleotide sequence length ≥500 bp (except for those made for *Celonites iranus* Gusenleitner, 2018, from which only a single sequence of 316 bp was obtained).

## 3. Results

### 3.1. Pollen-Collecting Apparatus of the Wasps

**Figure 2:**
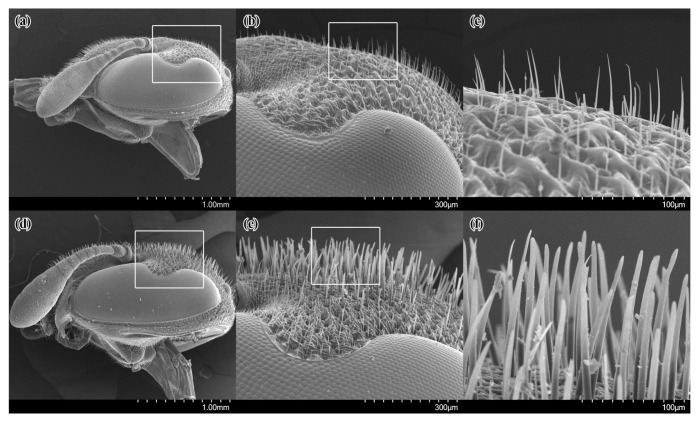
SEM micrographs of the head of females of *Celonites kozlovi* Kostylev, 1935 (**a**–**c**), and *C. sibiricus* Gusenleitner, 2007 (**d**–**f**), in lateral view: (**a**,**d**) overview; (**b**,**e**) close up of the frons showing setation (marked areas in **a**,**d**); (**c**,**f**) close up of the setae (marked areas in **b**,**e**).

In females of *Celonites kozlovi,* the frons is covered with thin, slightly ventro-medially curved setae with a delicate pointed tip that are about 50–60 μm long (Figure 2a–c). This is distinctly shorter than the diameter of the median ocellus.

**Figure 3:**
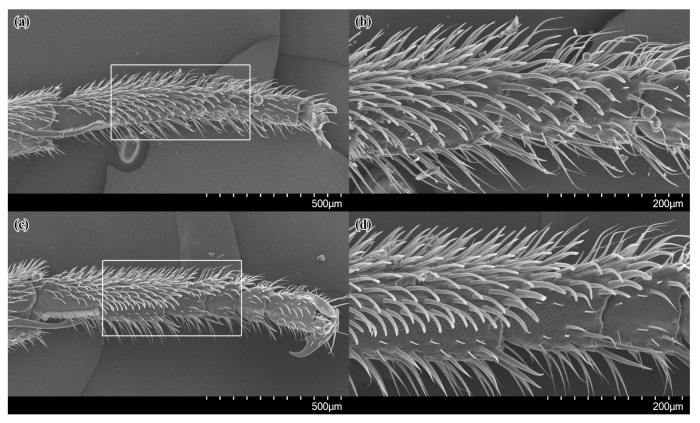
SEM micrographs of the inner side of the left fore-tarsus of females of *Celonites kozlovi* Kostylev, 1935 (**a**,**b**), and *C. sibiricus* Gusenleitner, 2007 (**c**,**d**): (**a**,**c**) overview; (**b**,**d**) close up of the distal half of the first, second, and third tarsomeres (marked areas in **a**,**c**).

The anterior margin and the adjacent anterior two-thirds of the inner side of the first and the second tarsomeres of the fore-tarsus are covered with stiff, thick setae that are slightly curved ventro-posteriorly, forming a pollen brush (Figure 3a,b). The setae are broad at their base, becoming gradually narrower towards their bluntly rounded distal end, which is weakly enlarged in some setae. These stiff setae are protruding over the nearly bare cuticula on the posterior third of the inner side of the first tarsomere and on the proximal base of the second tarsomere, which bears only a few small setae.

In females of *Celonites sibiricus*, the frons is covered with stiff, thick, flattened setae that are spatula-like with a rounded distal tip (Figure 2d–f). They are approximately 130 μm long, which is close to the diameter of the median ocellus. These setae are also located in the median area of the clypeus. The surface of the cuticula from which the stiff setae arise is very densely and finely wrinkled, resulting in a sharp-edged scaly structure.

The pollen brush on the fore-tarsus of the females of *Celonites sibiricus* is formed by stiff, thick setae that are quite similar to those of *C. kozlovi*, except that they are more parallel-sided and flattened distally, and their distal tip is more truncated (Figure 3c,d). On the first tarsomere, the pollen brush is delimited from the posteriorly adjacent area of nearly bare cuticula by a distinct row of more closely spaced, shorter setae. The area of bare cuticula is well defined, and nearly not protruded by the stiff setae of the pollen brush. It extends from the posterior third of the first over the second and third tarsomeres.

### 3.2. Flower-Visiting Records

**Figure 4:**
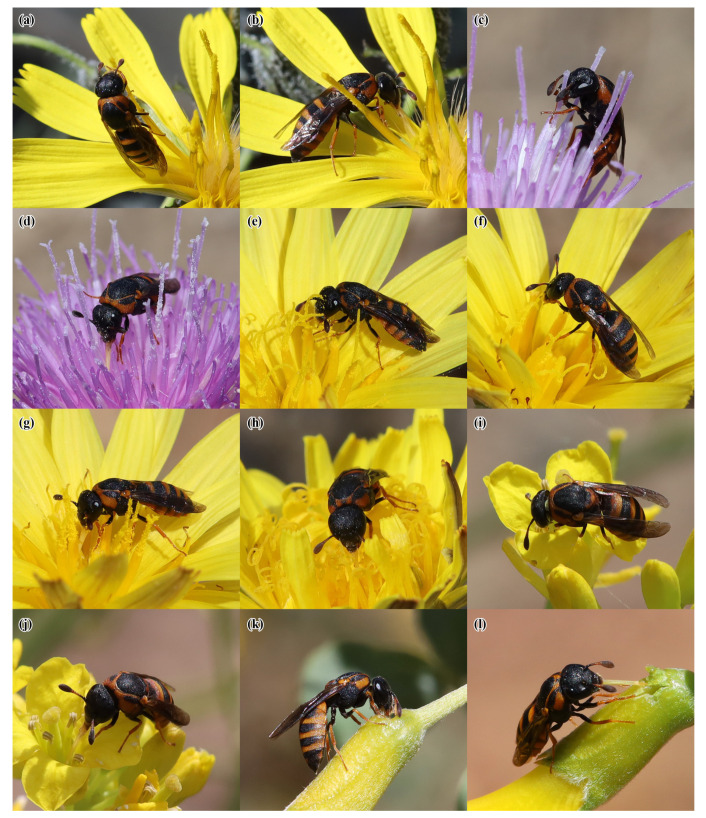
Females of *Celonites kozlovi* Kostylev, 1935, on flowers of various plant species: (**a**,**b**) *Crepidiastrum tenuifolium* (Willd.) Sennikov; (**c**,**d**) *Klasea marginata* (Tausch) Kitag.; (**e**–**g**) *Pseudopodospermum pubescens* (DC.) Zaika, Sukhor. & N. Kilian; (**h**) *Taraxacum* sp.; (**i**,**j**) *Sisymbrium polymorphum* (Murray) Roth; (**k**,**l**) *Caragana bungei* Ledeb. (**a**,**c**,**e**,**f**,**i**) pollen uptake; (**b**,**d**,**g**,**h**,**j**) regular nectar uptake; (**k**,**l**) nectar robbing.

**Figure 5:**
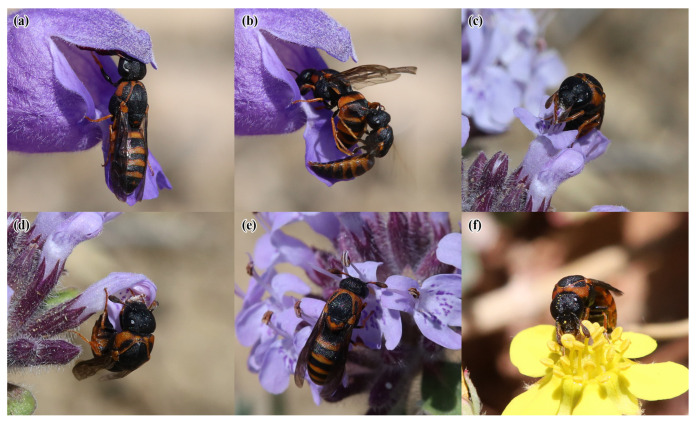
Females of *Celonites kozlovi* Kostylev, 1935, on flowers of various plant species: (**a**,**b**) *Dracocephalum peregrinum* L.; (**c**–**e**) *Ziziphora clinopodioides* Lam.; (**f**) *Sibbaldianthe bifurca* (L.) Kurtto & T. Erikss. (**a**,**c**,**d**,**f**) pollen uptake; (**b**) flower visit interrupted due to an encountering male; (**e**) nectar uptake.

**Figure 6:**
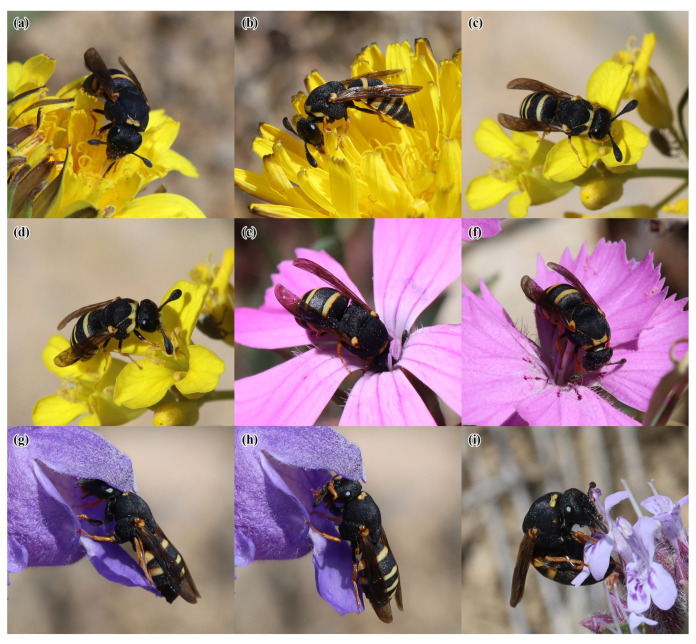
Females (**a**,**e**–**i**) and males (**b**–**d**) of *Celonites sibiricus* Gusenleitner, 2007, on flowers of various plant species: (**a**,**b**) *Taraxacum* sp.; (**c**,**d**) *Sisymbrium polymorphum* (Murray) Roth; (**e**,**f**) *Dianthus chinensis* L.; (**g**,**h**) *Dracocephalum peregrinum* L.; (**i**) *Ziziphora clinopodioides* Lam. (**a**,**b**,**d**,**f**) nectar uptake; (**c**,**e**,**g**,**h**) pollen uptake; (**i**) simultaneous nectar and pollen uptake.

A total of 40 plant species belonging to 18 families were recorded in flower during the observation period at the four study sites. *Celonites kozlovi* and *C. sibiricus* were recorded visiting flowers of 13 plant species from seven families (Table 2 and Figure 4, Figure 5 and Figure 6). Altogether 236 sightings (=first observations) on flowers were made, 185 of *C. kozlovi* and 51 of *C. sibiricus*. *Celonites kozlovi* was observed on flowers of 11 plant species belonging to seven families. Most sightings were made on the flowers of two species of Lamiaceae (85 sightings, 46%), followed by four species of Asteraceae (79 sightings, 43%). This indicates, that both families include principal forage plants of *C. kozlovi*, while visits to flowers of other families were less common (21 sightings, 11%). *Celonites sibiricus* was observed on flowers of seven plant species from five families. Two-thirds of the sightings were made on flowers of Lamiaceae (34 sightings, 67%), particularly of *Dracocephalum peregrinum* L. Visits of *C. sibiricus* to flowers of other plant families were occasional (17 sightings, 33%) and were mostly observed before *D. peregrinum* had started flowering in mid-June.

### 3.3. Behavior of the Wasps on Flowers

#### 3.3.1. Asteraceae

Females of *C. kozlovi* collected both pollen and nectar from flowers of three species of Asteraceae: *Crepidiastrum tenuifolium* (Willd.) Sennikov, *Klasea marginata* (Tausch) Kitag., and *Pseudopodospermum pubescens* (DC.) Zaika, Sukhor. & N. Kilian. On flowers of *C. tenuifolium,* females showed the following behavior. Each female approached a capitulum in flight and alighted on one of the spreading ligules. Then, she either moved to the nearest style (which is combined with the anthers in Asteraceae) to collect pollen (Figure 4a) or she extended her proboscis to reach the nectar hidden inside the floret (Figure 4b). During pollen uptake, the female stood on the style, holding on to it with the mid and hind legs, while the forelegs were used to brush pollen from the style (which typically carries the pollen grains aloft from the fused anthers that form a ring around the style close to its base) towards the mouthparts where it was consumed (Figure 4a).

The behavior of the wasps on flowers of *K. marginata* was only slightly different. Females alighted directly on disc florets, and then took up either pollen or nectar. During pollen uptake, the wasp stood on disc florets with her mid and hind legs, while the forelegs were used to brush pollen from the style towards the mouthparts. Pollen was consumed both from the inner surface of the fore-tarsi and directly from the style (Figure 4c), as well as from the cylinder of the fused anthers at its base. During nectar uptake, the female moved from one floret to another and inserted her proboscis into each corolla, holding on to the capitulum with all of her legs (Figure 4d). During a visit to a single capitulum, she usually alternated between pollen and nectar uptake.

The behavior of *C. kozlovi* females on flowers of *P. pubescens* differed from that at *C. tenuifolium* in that they were standing on a ligule during pollen uptake instead of holding on to the style due to the shorter styles of *P. pubescens*. The wasps also consumed pollen both directly from the style and the cylinder of the fused anthers (Figure 4e), as well as from the inner surface of their fore-tarsi, which were used to brush pollen towards the mouthparts (Figure 4f). The method of nectar uptake from flowers of *P. pubescens* was similar to that for *C. tenuifolium* (Figure 4g).

Females of both *C. kozlovi* and *C. sibiricus*, as well as males of the latter species, visited flowers of *Taraxacum* sp. This unidentified dandelion belongs to the section *Stenoloba* Kirschner & Štěpánek, all species of which are agamospermous [31]. According to our observations, this species did not produce any visible amount of pollen at all. Therefore, the wasps collected nectar only. They moved from one floret to another on the capitulum and merely inserted their proboscis into each floret (Figure 4h and Figure 6a,b).

#### 3.3.2. Brassicaceae

Females of *C. kozlovi* and *C. sibiricus*, as well as males of the latter species, were observed to visit flowers of *Sisymbrium polymorphum* (Murray) Roth. During nectar uptake the wasps usually inserted their proboscis into the flowers between the filaments while they held on to the petals with all of their legs (Figure 4j and Figure 6d). In addition, females of *C. kozlovi* were observed to ingest pollen directly from the anthers, while they held on to the petals and the stigma with their mid and hind legs (Figure 4i). A single male of *C. sibiricus* was observed to brush pollen from his clypeus and to consume it with the mouthparts after he had visited several flowers of *S. polymorphum* (Figure 6c).

#### 3.3.3. Caryophyllaceae

Two females of *C. sibiricus* were observed to have visited flowers of *Dianthus chinensis* L. In each case, the female visited only a single flower. The first female alighted on the petals and inserted her head as deep as possible into the flower so that the anthers appeared above its head (Figure 6e). The female then performed rapid back and forth movements of the anterior part of her body, rubbing her head over the anthers. This indicates pollen uptake. Another female visited a flower to take up nectar only. She inserted her proboscis deeply between the petals, while she held on to the stigma and filaments with all of her legs (Figure 6f).

Occasional visits by females of *C. kozlovi* and a male of *C. sibiricus* to flowers of *Gypsophila patrinii* Ser. were recorded. Detailed observations could not be made due to the very short duration of these visits, but at least nectar was probably collected.

#### 3.3.4. Convolvulaceae

Visits of a single female of *C. kozlovi* to flowers of *Convolvulus ammannii* Desr. were observed. The female inserted her proboscis into the corolla tube, indicating nectar uptake.

#### 3.3.5. Fabaceae

The only flowering shrub visited by pollen wasps in this study was *Caragana bungei* Ledeb. Females of *C. kozlovi* were observed on its flowers. They usually alighted on the calyx and inserted their proboscis into holes in the perianth (Figure 4k,l). These holes were not made by the pollen wasps themselves but had been gnawed by two species of eumenine wasps belonging to the genera *Onychopterocheilus* Blüthgen, 1955, and *Pterocheilus* Klug, 1805 (Hymenoptera: Vespidae: Eumeninae). Thus, the females of *C. kozlovi* acted as secondary nectar robbers, sensu Inouye [32]. Pollen uptake from a flower of *C. bungei* by a female of *C. kozlovi* was observed on a single occasion. In this case, the female alighted on the filaments of an open flower and consumed pollen from the anthers during a few seconds (the amount of pollen in the flower was low). It is of note that this female did not take up nectar in addition, although the calyx of the flower had a hole.

#### 3.3.6. Lamiaceae

On flowers of *Dracocephalum peregrinum,* the females of *Celonites kozlovi* alighted on the lower lip of the corolla. Then, they ingested pollen directly from the nototribic anthers with their mouthparts, which was usually supported by movements of the forelegs, while they were still standing on the lower lip of the corolla with their mid and hind legs (Figure 5a). After pollen consumption, the females moved head first deep into the corolla tube, where they remained until finally left the flower. Probably they took up nectar in this position, but the process was not visible from the outside. A male of *C. kozlovi* was observed on a single occasion to approach and disturb a female during her visit to a flower of *D. peregrinum* (Figure 5b). This female, however, immediately rejected the male and continued foraging.

During flower visits to *D. peregrinum,* each female of *C. sibiricus* also alighted on the lower lip of the corolla, but she took up pollen by rubbing the dorso-anterior parts of her head over the nototribic anthers (Figure 6g). In this manner, pollen grains were removed from the thecae and accumulated on the frons of the wasp. Rubbing over the anthers (Figure 6g) was regularly interrupted by behavioral phases in which the female brushed the pollen that had accumulated on frons and clypeus towards the mouthparts by alternating movements of her forelegs (Figure 6h). In the process, the inner side of each fore-tarsus was moved over the facial cuticula from dorsal to ventral areas. The mid and hind legs were always used to hold on to the corolla. After that, the female went deep into the corolla tube to take up nectar in the same way as *C. kozlovi* did. Males of *C. sibiricus* were observed at the flowers of *D. peregrinum* only during nectar uptake.

*Ziziphora clinopodioides* Lam. was visited by females of both *Celonites* species, which took up pollen as well as nectar from this plant. *Celonites kozlovi* consumed pollen directly from the anthers. This was usually supported by the forelegs, which were used to brush pollen towards the mouthparts (Figure 5c). Sometimes females took up pollen from flowers of *Z. clinopodioides* that were still not fully open (Figure 5d). In all cases, the mid and hind legs were used to hold on to the corolla of the flower. For nectar uptake, the wasps merely inserted their proboscis into the corolla tube (Figure 5e).

Females of *C. sibiricus* showed a different behavior. They alighted on the lower lip of the flower and took up nectar and pollen simultaneously. The proboscis was protruded deep into the corolla tube, while the female performed rapid back and forth movements of the anterior part of her body, rubbing her head over the nototribic anthers (Figure 6i). From time to time pollen was brushed from the frons to the mouthparts with the fore-tarsi by alternating movements of the forelegs, where it was finally ingested.

#### 3.3.7. Rosaceae

A single flower-visiting record is available for *Potentilla sericea* L., which was visited by a female of *C. sibiricus*. Due to the short duration of the visit, detailed observations could not be made, but at least nectar was probably collected.

Flowers of *Sibbaldianthe bifurca* (L.) Kurtto & T. Erikss. were sometimes visited by females of *C. kozlovi,* which collected both pollen and nectar. During pollen uptake the females were standing on the petals and consumed pollen directly from the anthers (Figure 5f). For nectar uptake, females protruded their proboscis and inserted it between the filaments.

### 3.4. DNA Barcoding Results: Taxon Tree

**Figure 7:**
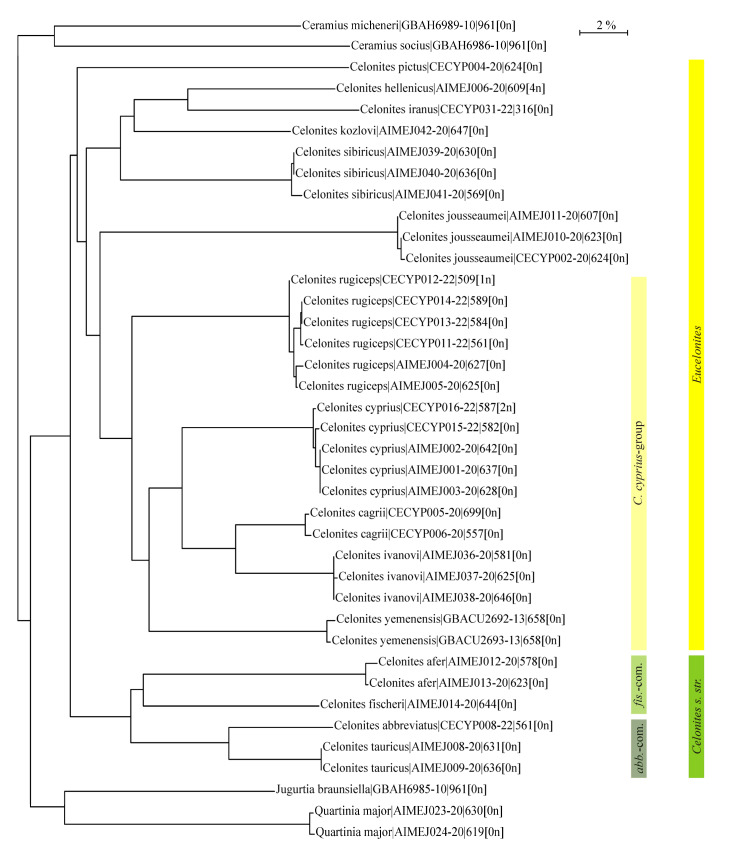
Neighbor-joining TaxonID tree of 15 Palaearctic species of *Celonites* and four species belonging to other genera of Masarini resulting from analyses of the barcoding sequences of the mitochondrial COI-5P gene of 39 individuals (labeled with species name|BOLD process ID|sequence length[ambiguous bases]; *abb.*-com. = *Celonites abbreviatus*-complex; *fis.*-com. = *C. fischeri*-complex).

Analysis of the barcoding sequences of the mitochondrial COI-5P gene of fifteen Palaearctic *Celonites* species together with four species belonging to the genera *Quartinia*, *Jugurtia* de Saussure, 1854, and *Ceramius* Latreille, 1810, as outgroups resulted in the neighbor-joining TaxonID Tree shown in Figure 7. The subgenera *Celonites* s. str. and *Eucelonites* Richards, 1962, represent well defined groups, as well as the *C. abbreviatus*-complex and the *C. fischeri*-complex. Within *Eucelonites*, *Celonites sibiricus* and *C. kozlovi* form a clade together with *C. hellenicus* Gusenleitner, 1997, and *C. iranus*.

## 4. Discussion

The only specialized structure for pollen uptake in females of *Celonites kozlovi* is a pollen brush on the first, second, and partly third tarsomeres of the foreleg. The setae forming this brush are broad and stiff, which allow them to withstand the mechanical forces that in all likelihood arise when they are moved over the anthers. The slightly curved form of the setae and their bluntly rounded tip may prevent the brush from getting stuck during brushing and probably increases the amount of pollen that can be removed. The existence of an unmodified pollen brush on the fore-tarsus is a character of the ground pattern of *Celonites,* which has already been adopted from the ground pattern of the Masarini [5,9]. The setation with thin, pointed setae on the frons of the females is also a plesiomorphic character, which is also present in the closely related *C. hellenicus* and *C. iranus* and in all other species of *Eucelonites* (except *C. sibiricus*) and the *C. fischeri*-complex of *Celonites* s. str. [5,17,33].

The plesiomorphic condition of the pollen-collecting apparatus is in congruence with the observed plesiomorphic pollen-collecting behavior of females of *C. kozlovi,* which consume pollen directly from the anthers, only supported by brushing movements of the forelegs from the pollen bearing structures of the flowers towards the mouthparts.

The unspecialized method of pollen uptake in *Celonites kozlovi* is reflected by its generalistic flower associations being recorded from 11 plant species belonging to seven families with very different flower types. Moreover, it has been observed to visit two additional species of Asteraceae before [23]. Pollen collection occurred on flowers of at least five different families, though the wasps were mainly associated with Lamiaceae and Asteraceae. Therefore, *C. kozlovi* is a polylectic species in the narrow sense, sensu Müller and Kuhlmann [24]. Polylecty is rare in *Celonites* and has only been proved for two Afrotropical species, which are *C. capensis* Brauns, 1905, and *C. promontorii* Brauns, 1905 [3,11]. Palaearctic species of the genus *Celonites* for which flower associations are known have so far only been found to be either broadly or narrowly oligolectic, sensu Müller and Kuhlmann [24], collecting pollen exclusively from flowers of Lamiaceae [18,26,33,34,35,36] or Boraginaceae [5,16,17,19,37]. Another noteworthy behavior of *C. kozlovi* is the consumption of pollen from flowers that have not yet fully opened. This strategy is also used by females of *C. fischeri* that consume pollen directly from the anthers as well [16]. Flowers in an early stage of flowering can be expected to offer larger amounts of pollen in comparison with those of older flowers, in which the pollen has already been depleted by other flower visitors. Direct pollen uptake enables *C. kozlovi* and *C. fischeri* to collect pollen from flowers before their anthers are accessible to other regular flower visitors.

The high plasticity of the flower-visiting behavior of *C. kozlovi* is likewise shown in its ability to rob nectar from already existing holes in the perianth of the Fabaceae *Caragana bungei*. Nectar robbing occurs in several species of eumenine wasps [38,39,40,41,42], especially those that use nectar as bonding agent during nest construction [40,41,42]. It is also common among bees [43], but it has not been reported for pollen wasps before. In this context, it is of note that in *Celonites,* nectar is also used as bonding agent during nest construction [2,3,16].

In females of *Celonites sibiricus,* the morphological structures on the front of the head and the fore-tarsi are adapted to indirect pollen uptake from nototribic anthers. The facial setation consists of stiff, spatula-like setae with rounded tips that are sufficient to withstand the mechanical forces, which arise when they are rubbed over the anthers, make it possible to remove pollen from the thecae. The wrinkled surface of the facial cuticula probably facilitates pollen grains adhering to it. The fore-tarsal pollen brush is also modified with a close row of setae along the posterior margin of the pollen brush, which is distinctly delimited from a posteriorly adjacent nearly bare area. Pollen grains are probably more efficiently removed from the facial pollen collecting area on frons and clypeus with the comb-like row of setae and accumulate in the bare area in front of the row, while the inner side of the tarsus is moved downwards over the facial setae. Hence, the structure is interpreted as an additional adaptation to pollen transfer from the exoskeleton to the mouthparts. This is consistent with the observation that more than two-thirds of the visits of the females of *C. sibiricus* occurred on nototribic flowers of Lamiaceae and the observed behavior of the females during indirect pollen uptake from these flowers. In another locality, *C. sibiricus* almost exclusively visited nototribic flowers of two species of *Dracocephalum* as well, namely *D. peregrinum* and *D. nutans* L. [23]. The behavior of the wasps on these flowers was similar to that observed on *D. peregrinum* in the recent study sites.

The derived pollen-collecting apparatus of *C. sibiricus* resembles the morphological structures for pollen uptake from nototribic flowers of Lamiaceae that are present in the members of the *C. abbreviatus*-complex [18,26,33,34], which take up pollen from these flowers in a similar way as *C. sibiricus* does [26,34,35,36]. However, there are distinct differences. Firstly, the setae on the frons of the species of the *C. abbreviatus*-complex are not flattened, but characteristically knobbed at the distal end [18,26,33,34,44]. Secondly, the bare area of cuticula on the fore-tarsus of these species extends over the entire inner side of the first, second, and third tarsomeres and it is concave. Anteriorly the bare area is bounded by a single row of closely spaced short and stout apically hooked setae, forming a distinct pollen-comb along the anterior margin of the first and the second tarsomeres [18,34]. These well-defined differences in the specific quality of the characters indicate that they evolved independently in both taxa. This convergent adaptation to pollen uptake from nototribic flowers of Lamiaceae in the stem lines of *C. sibiricus* and the *C. abbreviatus*-complex is also supported by the clearly separated positions of these taxa in the TaxonID tree. A convergent evolution of a functionally consistent pollen-collecting apparatus in the form of specialized setae on the front of the head as an adaptation to the uptake of pollen from nototribic flowers is present in several bee species as well [26].

Interspecific competition for floral resources between *Celonites kozlovi* and *C. sibiricus,* which coexist in the same habitats, is probably reduced by the different niche widths of the species. While *C. kozlovi* is a polylectic generalist, *C. sibiricus* is significantly more specialized and can be formally classified as a polylectic species with strong preference for Lamiaceae. However, visits by *C. sibiricus* to flowers of both *Sisymbrium polymorphum* and *Dianthus chinensis* are infrequent and limited to the time before the onset of the flowering period of *Dracocephalum*. Therefore, *C. sibiricus* should be considered as broadly oligolectic. The specialization in pollen uptake from nototribic flowers of Lamiaceae allows the females of *C. sibiricus* to visit flowers of *Dracocephalum* more rapidly than *C. kozlovi* can [23]. On the other hand, as a generalist species, *C. kozlovi* can switch to other pollen sources, if necessary, in particular, Asteraceae.

The high proportion of visits to Asteraceae by *C. kozlovi* is remarkable. In bees, it has been demonstrated that the utilization of Asteraceae pollen as larval provision requires special physiological adaptations [45,46]. As Asteraceae are common and yield high pollen rewards, selection should favor the evolution of these adaptations [45]. Indeed, Asteraceae host a large number of specialized bee taxa [24,47], but there are also several groups of masarine wasps belonging to the genera *Ceramius*, *Jugurtia*, and *Quartinia,* which are restricted to Asteraceae or show a marked preference for flowers of this family [2,3,48]. This suggests the existence of the same physiological constraints in pollen wasps. In *Celonites*, only five Afrotropical species were recorded from Asteraceae, and for two of them Asteraceae seem to be regular pollen hosts [3]. *Celonites kozlovi* is the first Palaearctic species of *Celonites* observed to use Asteraceae as a pollen source. Interestingly, the two only known flower-visiting records of *C. hellenicus*, which is a member of the same clade as *C. kozlovi* and *C. sibiricus*, are also from Asteraceae (a male and a female on *Pallenis spinosa* (L.) Cass., according to an unpublished observation by V.M.). Moreover, females and males of *Celonites sibiricus* were sporadically observed to take up nectar from *Taraxacum* belonging to Asteraceae as well.

Other pollen sources of the polylectic *Celonites kozlovi* are also noteworthy. Members of the family Brassicaceae have not been recorded as forage plants of the masarine wasps before, and Caryophyllaceae, Fabaceae, and Rosaceae are reported as pollen sources of the genus *Celonites* for the first time, according to the previously published data [2,3,5,11,12,13,16].

## 5. Conclusions

*Celonites kozlovi* and *C. sibiricus* are closely related, but demonstrate two different foraging strategies. The first species is polylectic in a narrow sense and collects pollen from flowers of plants belonging to several families (with the predomination of Asteraceae and Lamiaceae) and various flower types using rather diverse methods of both pollen and nectar uptake. This is possible due to a high degree of behavioral plasticity. The generalistic foraging strategy of *C. kozlovi* is correlated with an unspecialized, plesiomorphic pollen-collecting apparatus. In contrast, *Celonites sibiricus* is broadly oligolectic and collects pollen predominately from flowers of Lamiaceae. This specialized foraging strategy is associated with apomorphic behavioral and morphological traits that allow the females to collect pollen from the nototribic anthers of these flowers more rapidly. The adaptations to pollen uptake from nototribic flowers in *C. sibiricus* evolved independently to a similar specialization process in the stem line of the *C. abbreviatus*-complex. Perhaps, the existence of two different foraging strategies in the closely related species leads to niche segregation and allows them to sympatrically coexist in the same habitats in the mountain semi-desert of the Altai Mountains.

## Figures and Tables

**Table 1:** Study sites of *Celonites kozlovi* Kostylev, 1935, and *C. sibiricus* Gusenleitner, 2007, in the Altai Mountains and observation time in 2022.

Locality Name	Latitude	Longitude	Date	Duration of Observations (hours)
Tydtuyaryk River valley	50°04′25″ N	88°25′12″ E	12.VI.2022	1
			18.VI.2022	2
			21.VI.2022	2
			22.VI.2022	2
			23.VI.2022	1
			26.VI.2022	0.25
Mars Natural Landmark	50°03′50″ N	88°18′45″ E	17.VI.2022	0.75
			23.VI.2022	1.5
			25.VI.2022	0.5
3 km to the north-east of Kokorya	49°56′31″ N	89°02′45″ E	24.VI.2022	0.5
5 km to the north-east of Kokorya	49°57′00″ N	89°04′19″ E	24.VI.2022	0.5
			26.VI.2022	1
Total				13

**Table 2:** Flower-visiting records of *Celonites kozlovi* Kostylev, 1935, and *C. sibiricus* Gusenleitner, 2007, from the Altai Mountains during 13 h of random transect walks in 2022.

Plant Taxon	Σ Sightings of Flower-Visiting Individuals			
	*C. kozlovi*		*C. sibiricus*	
	♀	♂	♀	♂
Asteraceae				
*Crepidiastrum tenuifolium* (Willd.) Sennikov	2			
*Klasea marginata* (Tausch) Kitag.	41	1		
*Pseudopodospermum pubescens* (DC.) Zaika, Sukhor. & N. Kilian	26			
*Taraxacum* sp.	10		2	2
Brassicaceae				
*Sisymbrium polymorphum* (Murray) Roth	7		3	6
Caryophyllaceae				
*Dianthus chinensis* L.			2	
*Gypsophila patrinii* Ser.	2			1
Convolvulaceae				
*Convolvulus ammannii* Desr.	1			
Fabaceae				
*Caragana bungei* Ledeb.	6			
Lamiaceae				
*Dracocephalum peregrinum* L.	66		24	9
*Ziziphora clinopodioides* Lam.	18		1	
Rosaceae				
*Potentilla sericea* L.			1	
*Sibbaldianthe bifurca* (L.) Kurtto & T. Erikss.	5			
Other plant taxa *				
Total	184	1	33	18

* Other plant taxa in flower were the following: Apiaceae: *Ferula caspica* M. Bieb., *F. songarica* Pall. ex Willd.; Asteraceae: *Aster alpinus* L., *Galatella altaica* Tzvelev; Boraginaceae: *Eritrichium pulviniforme* Popov, *Lappula* sp.; Brassicaceae: *Lepidium amplexicaule* Willd., *L. cordatum* Willd. ex Steven, *Ptilotrichum canescens* (DC.) C.A. Mey.; Caryophyllaceae: *Stellaria dichotoma* L.; Chenopodiaceae: *Chenopodium frutescens* C.A. Mey.; Fabaceae: *Caragana pygmaea* (L.) DC., *Oxytropis pumila* Fisch. ex DC., *Vicia costata* Ledeb.; Lamiaceae: *Nepeta annua* Pall., *Panzerina canescens* (Bunge) Soják, *P. lanata* (L.) Soják; Nitrariaceae: *Nitraria sibirica* Pall.; Orobanchaceae: *Orobanche coerulescens* Stephan; Plumbaginaceae: *Goniolimon speciosum* (L.) Boiss.; Polygonaceae: *Atraphaxis pungens* (M. Bieb.) Jaub. & Spach, *Rheum compactum* L.; Santalaceae: *Thesium* sp.; Ranunculaceae: *Thalictrum acutilobum* DC.; Rosaceae: *Cotoneaster laxiflorus* J. Jacq. ex Lindl.; Rubiaceae: *Galium verum* L.; Zygophyllaceae: *Zygophyllum pinnatum* Cham.

**Figure A1:**
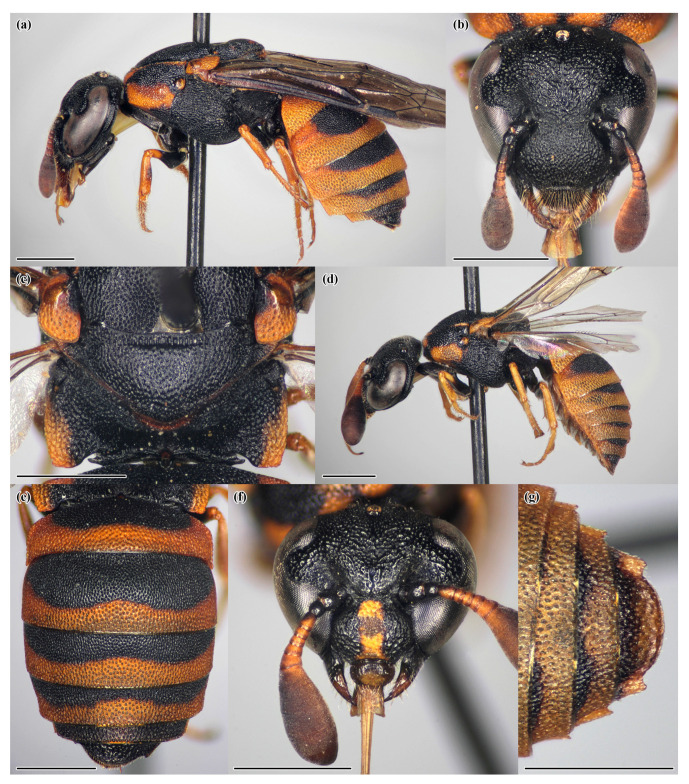
Female (**a**–**c**,**e**) and male (**d**,**f**,**g**) of *Celonites kozlovi* Kostylev, 1935: (**a**,**d**) habitus in lateral view; (**b**,**f**) head in frontal view; (**c**) posterior half of the mesosoma in dorsal view; (**e**) metasoma in dorsal view; (**g**) posterior end of the metasoma in dorsal view. Scale bars = 1 mm.

**Figure A2:**
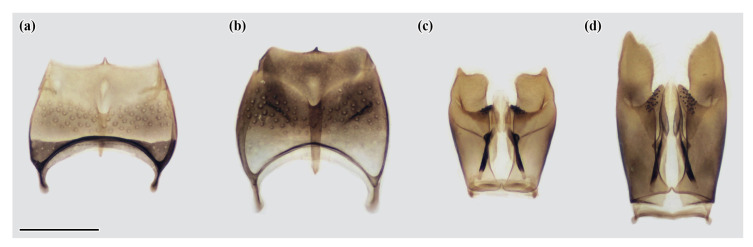
*Celonites kozlovi* Kostylev, 1935 (**a**,**c**) and *C. sibiricus* Gusenleitner, 2007 (**b**,**d**): (**a**,**b**) male sternum VII + VIII in ventral view; (**c**,**d**) male genitalia in dorsal view. Scale bar = 0.5 mm.

## Data Availability

The data presented in this study are available in the article and the Supplementary Materials. All nucleotide sequences were uploaded and stored in the BOLD database (www.boldsystems.org, accessed on 14 February 2023).

## Supplementary Materials

The following supporting information can be downloaded at: https://www.mdpi.com/article/10.3390/insects14050408/s1, Table S1: The data on the specimens used for barcoding sequences of the mitochondrial COI-5P gene.

## Appendix A. Re-Description of *Celonites kozlovi* Kostylev, 1935

Female (Figure A1a–c,e): Body length (from head to posterior margin of tergum II) 4.5–6.0 mm; fore wing length 4.5–5.5 mm. Head in frontal view about 1.2× as wide as long (from top of median ocellus to ventral margin of clypeus). Clypeus about 1.4× as wide as long; its ventro-medial emargination shallow, about 0.2× as deep as wide, taking about 1/2 of clypeal width, apical teeth blunt. Distance between lateral ocellus and occiput about 0.8× as long as distance between lateral ocelli. Antenna with articles A8–A12 forming ventrally flattened club about 2.0 times as long as wide in dorsal view. Maximal width of gena slightly exceeding diameter of median ocellus. Preoccipital carina distinct. Pronotum with anterior side nearly vertical, roundly angled to dorsal surface. Anterior pronotal carina, sensu Carpenter [8], rather weak. Posterior pronotal carina forms low narrow translucent sinuate crest on humeral angle of pronotum. Scutellum separated from mesoscutum by broad transverse suture, slightly convex, bluntly angled posteriorly. Axilla of scutellum without distinct lateral projection. Epipleural and episternal sulci weak. Mesepimeron produced postero-laterally as small blunt tubercle. Epicnemial carina distinct but not sharp. Outline of emargination between lateral lamella and postero-lateral process of propodeum with narrow short neck, its apical end medially enlarged. Terga I–V medially with straight, not crenulate margins, laterally with tooth. Tergum VI bluntly angulate posteriorly, laterally with tooth. Sternum VI postero-medially with short and acute process.

Clypeus densely wrinkly-punctate; interstices much less than puncture diameter, matt. Frons with more distinct wrinkled sculpture, medially often with weak longitudinal furrow between wrinkles; interstices shining. Vertex and gena densely punctate; interstices less than puncture diameter, matt, with distinctly shagreened microsculpture. Pronotum, scutum, and scutellum densely reticulately-punctate, punctures larger than those on vertex; interstices less than puncture diameter, matt, with distinctly shagreened microsculpture. Tegula deeply punctate, punctures smaller than those on pronotum, scutum, and scutellum; interstices matt, about as puncture diameter, with distinctly shagreened microsculpture. Dorsal and ventral mesepisterna and mesepimeron sculptured similarly to pronotum, scutum, and scutellum but punctures somewhat larger. Posterior surface of metanotum, metapleuron, and lateral surface of propodeum matt, with indistinct sculpture. Posterior surface of propodeum longitudinally wrinkled; postero-lateral process of propodeum densely reticulately-punctate; interstices much less than puncture diameter, matt, with distinctly shagreened microsculpture. Punctation on terga very dense and deep, particularly on disc where interstices much less than puncture diameter, matt, with distinctly shagreened microsculpture. Depression with similar but sparser punctures resembling those on tegula. Sterna I–V with scattered shallow punctures; interstices exceed puncture diameter, matt, with reticulate microsculpture. Sternum VI medially with shining impunctate line continuing to posterior process; elsewhere with deep and very large punctures; interstices less than puncture diameter, rather shining, with scattered micropunctures.

Clypeus and frons covered with thin setae which are shorter than diameter of median ocellus. Mandible and labrum with thicker and somewhat longer setae, about as long as pedicel length. Outer surface of tibiae with short stiff setae; inner surface of tibia and tarsi with rather long stiff setae. Other parts of body bare or with minute unnoticeable setae.

Black with variable ferruginous pattern. Distal half of mandible ferruginous. Clypeus usually black but sometimes basally with ferruginous central spot. Antenna mostly darkened dorsally but sometimes articles A3–A7 or rarely entire flagellum ferruginous; ventral surface of flagellum ferruginous. Pronotum from nearly entirely black to nearly entirely ferruginous. Tegula mostly ferruginous. Scutellum often with irregular ferruginous pattern on posterior half and on axilla. Metanotum often with ferruginous spot at center. Dorsal mesepisternum sometimes with irregular ferruginous spot anteriorly. Postero-lateral process of propodeum usually with ferruginous spot laterally. Terga I–V or at least terga I–IV with apical ferruginous bands or at least apical central spots; ferruginous pattern of various degree of development but confined to depression and lateral parts of disc. Sterna I–III from entirely black to entirely ferruginous, usually black with spot postero-laterally on each side. Sterna IV–V entirely black or with small ferruginous spot postero-laterally on each side. All legs ferruginous from ends of femora.

Male (Figure A1d,f,g and Figure A2a,c): Body length (from head to posterior margin of tergum II) 4.5–5.5 mm; forewing length 4.5–5.0 mm. Structure resembles that of female but head in frontal view about 1.3× as wide as long (from top of median ocellus to ventral margin of clypeus); clypeus about 1.3× as wide as long; distance between lateral ocellus and occiput about 0.9× as long as distance between lateral ocelli. Ventral side of antennal club with three tyloids on articles A9, A10, and A11; tyloid of A11 smaller than others. Tergum VI and sternum VI as those of preceding segment. Tergum VII broadly rounded medio-posteriorly as in *C. sibiricus* [22], laterally with tooth. Sternum VII + VIII as in Figure A2a, shorter than that of *C. sibiricus* (Figure A2b), with deep pit at center and small process medially at apical margin. Male genitalia as in Figure A2c, shorter than those of *C. sibiricus* (Figure A2d).

Sculpture and setation as in female. Coloration resembles that of female, but labrum at least partially orange-yellow and clypeus with orange-yellow medial band which can be either reduced to ventral central spot or enlarged to nearly entire clypeus. All terga with ferruginous posterior bands and all sterna entirely ferruginous.

## Appendix B. Collected Material

*Celonites kozlovi* Kostylev, 1935. RUSSIA: *Altai Republic*: Tydtuyaryk Riv. vall., 50°04′25″ N, 88°25′12″ E, 12.VI.2022, 1 ♀, leg. A. Fateryga; ibid., on *Klasea marginata,* 12.VI.2022, 1 ♂, leg. A. Fateryga; ibid., 12.VI.2022, 5 ♀, leg. M. Proshchalykin; ibid., 18.VI.2022, 5 ♀, leg. M. Proshchalykin; ibid., 21.VI.2022, 12 ♀, 1 ♂, leg. M. Proshchalykin; ibid., 22.VI.2022, 2 ♀, leg. M. Proshchalykin; “Mars”, 50°03′50″ N, 88°18′45″ E, 17.VI.2022, 2 ♀, leg. M. Proshchalykin; ibid., 22.VI.2022, 2 ♀, leg. M. Proshchalykin; ibid., 23.VI.2022, 2 ♀, leg. M. Proshchalykin; ibid., 25.VI.2022, 1 ♂, leg. A. Fateryga; ibid., 25.VI.2022, 2 ♀, leg. M. Proshchalykin; 5 km NE Kokorya, 49°57′00″ N, 89°04′19″ E, on *Caragana bungei,* 26.VI.2022, 1 ♀, leg. A. Fateryga.

*Celonites sibiricus* Gusenleitner, 2007. RUSSIA: *Altai Republic*: Tydtuyaryk Riv. vall., 50°04′25″ N, 88°25′12″ E, 14.VI.2022, 1 ♀, leg. M. Proshchalykin; ibid., 18.VI.2022, 1 ♀, leg. M. Proshchalykin; ibid., 21.VI.2022, 3 ♀, leg. M. Proshchalykin; ibid., 26.VI.2022, 1 ♂, leg. M. Proshchalykin; “Mars”, 50°03′50″ N, 88°18′45″ E, 17.VI.2022, 1 ♂, leg. A. Fateryga; ibid., on *Taraxacum* sp.*,* 17.VI.2022, 1 ♀, leg. A. Fateryga; ibid., on *Sisymbrium polymorphum,* 17.VI.2022, 1 ♂, leg. A. Fateryga; ibid., 17.VI.2022, 1 ♂, leg. M. Proshchalykin; ibid., on *Dracocephalum peregrinum,* 23.VI.2022, 1 ♂, leg. A. Fateryga; 5 km NE Kokorya, 49°57′00″ N, 89°04′19″ E, 24.VI.2022, 1 ♂, leg. A. Fateryga; ibid., on *Gypsophila patrinii,* 26.VI.2022, 1 ♂, leg. M. Proshchalykin; 15 km SE Kuray, 50°11′10″ N, 88°07′04″ E, 15.VI.2022, 2 ♂, leg. A. Fateryga; ibid., 15.VI.2022, 1 ♀, leg. M. Proshchalykin; ibid., 17.VI.2022, 6 ♀, leg. M. Proshchalykin; ibid., 21.VI.2022, 2 ♀, leg. M. Proshchalykin.

## References

[1] Rahmani Z., Rakhshani E., Carpenter J.M. (2020). Updated checklist of Vespidae (Hymenoptera: Vespoidea) in Iran. J. Insect Biodivers. Syst..

[2] Gess S.K. (1996). The Pollen Wasps. Ecology and Natural History of the Masarinae.

[3] Gess S.K., Gess F.W. (2010). Pollen Wasps and Flowers in Southern Africa. SANBI Biodiversity Series 18.

[4] Carpenter J.M., Garcete-Barrett B.R., Hermes M.G. (2006). Catalog of the Neotropical Masarinae (Hymenoptera, Vespidae). Rev. Bras. Entomol..

[5] Richards O.W. (1962). A Revisional Study of the Masarid Wasps (Hymenoptera, Vespoidea).

[6] Mauss V., Kuba K., Krenn H.W., Krenn H.W. (2019). Evolution of the multifunctional mouthparts of adult Vespidae. Insect Mouthparts. Zoological Monographs 5.

[7] Carpenter J.M. (1982). The phylogenetic relationship and natural classification of the Vespoidea (Hymenoptera). Syst. Entomol..

[8] Carpenter J.M. (1988). The phylogenetic system of the Gayellini (Hymenoptera: Vespidae; Masarinae). Psyche.

[9] Mauss V. (2007). Evolution verschiedener Lebensformtypen innerhalb basaler Teilgruppen der Faltenwespen (Hymenoptera, Vespidae). Denisia.

[10] Piekarski P.K., Carpenter J.M., Lemmon A.R., Lemmon E.M., Sharanowski B.J. (2018). Phylogenomic evidence overturns current conceptions of social evolution in wasps (Vespidae). Mol. Biol. Evol..

[11] Gess S.K., Gess F.W. (1989). Flower visiting by masarid wasps in southern Africa (Hymenoptera: Vespoidea: Masaridae). Ann. Cape Prov. Mus. Nat. Hist..

[12] Gess S.K., Gess F.W., Gess R.W. (1997). Update on the flower associations of southern African Masarinae with notes on the nesting of *Masarina strucki* Gess and *Celonites gariepensis* Gess (Hymenoptera: Vespidae: Masarinae). J. Hymenopt. Res..

[13] Gess S.K., Gess F.W. (2004). Distributions of flower associations of pollen wasps (Vespidae: Masarinae) in southern Africa. J. Arid Environ..

[14] Carpenter J.M. (2001). Checklist of species of the subfamily Masarinae (Hymenoptera: Vespidae). Amer. Mus. Novit..

[15] Fateryga A.V., Fadeev K.I. The identity of *Celonites montanus* Mocsáry, 1906 (Hymenoptera: Vespidae: Masarinae) and its first record from Kazakhstan. Zootaxa.

[16] Mauss V., Müller A. (2014). First contribution to the bionomics of the pollen wasp *Celonites fischeri* Spinola, 1838 (Hymenoptera, Vespidae, Masarinae) in Cyprus. J. Hymenopt. Res..

[17] Mauss V., Fateryga A.V., Yildirim E., Carpenter J.M. (2022). Contribution to the taxonomy, bionomics and distribution of the Palaearctic *Celonites cyprius*-group (Hymenoptera, Vespidae, Masarinae) with the description of two new species from the North Caucasus and East Anatolia. J. Hymenopt. Res..

[18] Fateryga A.V., Mauss V., Fateryga V.V. (2022). New distributional records of *Celonites tauricus* (Hymenoptera, Vespidae, Masarinae) and new data on its behaviour at flowers. J. Hymenopt. Res..

[19] Mauss V., Praz C.J., Müller A., Prosi R., Rosa P. (2022). Description of the nest of the pollen wasp *Celonites jousseaumei* Du Buysson, 1906 (Hymenoptera, Vespidae, Masarinae) with a new host association of the cuckoo wasp *Spintharina innesi* (Du Buysson, 1894) (Hymenoptera, Chrysididae). J. Hymenopt. Res..

[20] Kostylev G. (1935). Materialien zur Kenntnis der Masariden-Fauna der Paläarktis. Arch. Muz. Zool. Univ. Moscou.

[21] Gusenleitner J. (1991). Über Vespoidea (Hymenoptera) aus der Mongolei und der Sovietunion. Linz. Biol. Beitr..

[22] Gusenleitner J. (2007). Eine neue *Celonites*-Art aus Sibirien (Hymenoptera: Vespidae, Masarinae). Linz. Biol. Beitr..

[23] Fateryga A.V. (2020). New records of *Celonites kozlovi* Kostylev, 1935 and *C. sibiricus* Gusenleitner, 2007 (Hymenoptera: Vespidae: Masarinae), with observations on their behavior at flowers. Far East. Entomol..

[24] Müller A., Kuhlmann M. (2008). Pollen hosts of western palaearctic bees of the genus *Colletes* (Hymenoptera: Colletidae): The Asteraceae paradox. Biol. J. Linn. Soc..

[25] Faegri K., van der Pijl L. (1979). The Principles of Pollination Ecology.

[26] Müller A. (1996). Convergent evolution of morphological specializations in Central European bee and honey wasp species as an adaptation to the uptake of pollen from nototribic flowers (Hymenoptera, Apoidea and Masaridae). Biol. J. Linn. Soc..

[27] Seregin A.P., Seregin A.P. (2023). Moscow University Herbarium (MW). Occurrence Dataset.

[28] Krasnoborov I.M., Artemov I.A., Krasnoborov I.M., Artemov I.A. (2012). Key to Plants of the Altai Republic.

[29] Plantarium Plants and Lichens of Russia and Neighboring Countries: Open Online Galleries and Plant Identification Guide. http://www.plantarium.ru/.

[30] POWO (2021). Plants of the World Online.

[31] Kirschner J., Štěpánek J. (2011). Dandelions in Central Asia: A revision of *Taraxacum* section *Stenoloba*. Preslia.

[32] Inouye D.W. (1980). The terminology of flower larceny. Ecology.

[33] Mauss V. (2013). Description of *Celonites andreasmuelleri* sp. n. (Hymenoptera, Vespidae, Masarinae) from the Middle East with a key to the *Palaearctic species* of the *C. abbreviatus*-complex of the subgenus *Celonites* s. str. J. Hymenopt. Res..

[34] Schremmer F. (1959). Der bisher unbekannte Pollensammelapparat der Honigwespe *Celonites abbreviatus* Vill. (Vespidae, Masarinae). Z. Morphol. Ökol. Tiere.

[35] Mauss V. (2006). Observations on flower associations and mating behaviour of the pollen wasp species *Celonites abbreviatus* (Villers, 1789) in Greece (Hymenoptera: Vespidae, Masarinae). J. Hymenopt. Res..

[36] Mauss V., Fateryga A.V., Prosi R. (2016). Taxonomy, distribution and bionomics of *Celonites tauricus* Kostylev, 1935, stat. n. (Hymenoptera, Vespidae, Masarinae). J. Hymenopt. Res..

[37] Popov V.B. (1948). Oligotrophism of species of the genus *Quartinia* Grib. (Hymenoptera, Vespoidea). Zool. Zhurnal.

[38] Haeseler V. (1980). Zum Necktarraub solitärer Faltenwespen (Hymenoptera: Vespoidea: Eumenidae). Entomol. Gen..

[39] Haeseler V. (1997). *Ancistrocerus oviventris* (Wesmael 1836), eine weitere Nektar raubende solitäre Faltenwespe (Hymenoptera: Vespoidea: Eumenidae). Faun.-Ökol. Mitt..

[40] Fateryga A.V., Podunay Y.A. (2018). Nesting and biology of *Alastor mocsaryi* (Hymenoptera, Vespidae: Eumeninae). Entomol. Rev..

[41] Fateryga A.V., Popovich A.V., Podunay Y.A., Fateryga V.V. (2020). First data on the bionomics of *Leptochilus* (*Euleptochilus*) *limbiferus* (Morawitz, 1867) (Hymenoptera: Vespidae: Eumeninae), with taxonomic notes and new records. Zootaxa.

[42] Fateryga A.V. (2021). Two new Nearctic genera in the tribe Odynerini s. str. revealed on the bionomics and morphology, with a comment on the cocoons of the eumenine wasps (Hymenoptera: Vespidae: Eumeninae). Far East. Entomol..

[43] Müller A., Westrich P. (2023). Morphological specialisation for primary nectar robbing in a pollen specialist mining bee (Hymenoptera, Andrenidae). J. Hymenopt. Res..

[44] Mauss V., Prosi R. (2018). Identity and distribution of *Celonites hermon* Gusenleitner, 2002 (Hymenoptera, Vespidae, Masarinae) from the Middle East with a description of the hitherto unknown male. J. Hymenopt. Res..

[45] Praz C.J., Müller A., Dorn S. (2008). Specialized bees fail to develop on non-host pollen: Do plants chemically protect their pollen?. Ecology.

[46] Sedivy C., Müller A., Dorn S. (2011). Closely related pollen generalist bees differ in their ability to develop on the same pollen diet: Evidence for physiological adaptations to digest pollen. Funct. Ecol..

[47] Müller A. (1996). Host-plant specialization in Western Palearctic anthidiine bees (Hymenoptera: Apoidea: Megachilidae). Ecol. Monogr..

[48] Mauss V., Müller A., Prosi R. (2018). Flower associations and nesting of the pollen wasp *Quartinia major* Kohl, 1898 (Hymenoptera, Vespidae, Masarinae) in Morocco. J. Hymenopt. Res..

